# Fast and easy bioassay for the necrotizing fungus *Botrytis cinerea* on poplar leaves

**DOI:** 10.1186/s13007-023-01011-3

**Published:** 2023-03-29

**Authors:** Steven Dreischhoff, Ishani Shankar Das, Felix Häffner, Anna Malvine Wolf, Andrea Polle, Karl Henrik Kasper

**Affiliations:** 1 Forest Botany and Tree Physiology, University of Goettingen, 37077 Göttingen, Germany; 2 Department Aquatic Ecosystem Analysis, Helmholtz Center for Environmental Research-UFZ, Magdeburg, Germany; 3 Hessische Landesgesellschaft mbh, Gießen, Germany

**Keywords:** *Botrytis cinerea*, Poplar, Necrosis, Pathogen, Fungus, Quantification, Jasmonate, *Populus* spec., Infection assay

## Abstract

**Background:**

Necrotizing pathogens pose an immense economic and ecological threat to trees and forests, but the molecular analysis of these pathogens is still in its infancy because of lacking model systems. To close this gap, we developed a reliable bioassay for the widespread necrotic pathogen *Botrytis cinerea* on poplars (*Populus* sp.), which are established model organisms to study tree molecular biology.

**Results:**

*Botrytis cinerea* was isolated from *Populus* x *canescens* leaves*.* We developed an infection system using fungal agar plugs, which are easy to handle. The method does not require costly machinery and results in very high infection success and significant fungal proliferation within four days. We successfully tested the fungal plug infection on 18 poplar species from five different sections. Emerging necroses were phenotypically and anatomically examined in *Populus* x *canescens* leaves. We adapted methods for image analyses of necrotic areas. We calibrated *B. cinerea* DNA against Ct-values obtained by quantitative real-time polymerase chain reaction and measured the amounts of fungal DNA in infected leaves. Increases in necrotic area and fungal DNA were strictly correlated within the first four days after inoculation. Methyl jasmonate pretreatment of poplar leaves decreased the spreading of the infection.

**Conclusions:**

We provide a simple and rapid protocol to study the effects of a necrotizing pathogen on poplar leaves. The bioassay and fungal DNA quantification for *Botrytis cinerea* set the stage for in-depth molecular studies of immunity and resistance to a generalist necrotic pathogen in trees.

**Supplementary Information:**

The online version contains supplementary material available at 10.1186/s13007-023-01011-3.

## Background

*Botrytis cinerea* is the anamorph of *Botryotinia fuckeliana* and the causal agent of grey mold disease [1]. It is one of the most widespread pathogens threatening the health of over 1400 species in the plant kingdom [2, 3]. *B. cinerea* infections destroy fruits, field crops, shrubs, and trees [1], causing annual economic losses of up to $100 billion worldwide [4]. The airborne disease can remain symptomless within a plant until favorable conditions for the outbreak of the infection prevail [5].

Often overshadowed by its tremendous impact on crop production, it has been overlooked that *B. cinerea* is also a major problem for many forest tree species [6, 7]. Büsgen [8] described necroses caused by *B. cinerea* on leaves of several broadleaf tree species, such as *Ulmus montana*, *Populus alba*, *Tilia parvifolia,* and *Prunus avium*. Seedlings and saplings of economically important tree species such as *Abies* spp., *Pinus* ssp., *Cupressus* ssp., *Larix* ssp., *Tsuga* ssp., and *Cornus florida* can be damaged or killed by *B. cinerea* [9, 10]. *B. cinerea* does not show distinct habitat specificity and is endemic in boreal and hot and dry environments [11]. These conditions stress many tree species in the northern hemisphere, rendering them more susceptible to pathogen infections with the progression of climate change [12].

Poplar species are model organisms to study tree-pathogen interactions [13] and are widely used for biomass production [14, 15]. Modern protocols are available to research poplar interactions with biotrophic pathogens, such as *Melampsora larici-populina* [16]. Poplars employ salicylic acid-dependent defense pathways to fend off biotrophic fungal diseases, e.g., [16, 17]. Extensive studies in herbaceous plants such as *Arabidopsis thaliana* and arable crops [18, 19] showed that defense against necrotizing pathogens is regulated via jasmonate-dependent signaling pathways. To date, no state-of-the-art protocol is available for research on the interaction between poplar and necrotizing pathogens. Instead, even recent studies rely on the quantification of lesion area as a proxy for infection severity [20], a method prone to errors and uncertainties [21].

Here, we report the isolation of the potentially necrotizing pathogen *B. cinerea* from *Populus* x *canescens.* We present a fast and reliable protocol to infect various poplar species. We optimized fungal DNA extraction and quantification. The protocol uses the fast growth of *B. cinerea* under controlled conditions, its rapid infection cycle under optimal conditions [22], and the availability of highly specific qRT-PCR primers [23]. We demonstrate a correlation between necrotic leaf area and fungal DNA in the leaf. We conducted histological and anatomical analyses of necrotic and healthy leaf structures.

## Methods

### Origin and cultivation of poplar species

In vitro cultures of *P*. x *canescens* (INRA 717 1B4) and *P*. *euphratica* (clone B2 obtained from trees grown in the Ein Avdat region, Israel) were micropropagated as described by Müller et al. [24]. After four weeks of growth and rooting, the plantlets were potted in 3 L pots containing complete soil (Fruhstorfer Erde Type N, Hawita Gruppe, Vechta, Germany) and covered individually with transparent plastic beakers. To acclimate the plants to greenhouse conditions, the beakers were gradually lifted in the second week and removed at the beginning of the third week. The plants were cultivated in the greenhouse for five to eight weeks under ambient light with supplemental illumination of 150 µE m^−2^ s^−1^ photosynthetically active radiation with a 16/8 h day/night rhythm. The temperature ranged from 15 °C to 30 °C, and air humidity was between 53 and 82%. The plants were watered regularly with tap water.

Twigs (length: about 30–40 cm) were collected (13.04.2022, after bud break) from different poplar species grown in the Forest Botanical Garden (location: 51° 34’N 9° 57’O; mean annual precipitation sum: 624 mm; mean annual temperature: 9.2 °C). The twigs were placed in the greenhouse with the cut end submerged in tap water and kept for four days under greenhouse conditions as described above.

### Isolation and cultivation of *Botrytis cinerea*

Fourteen leaves of 12-week-old greenhouse-cultivated *P*. x *canescens* were cut and surface sterilized with 70% ethanol. The leaves were three times sterilized for five seconds, the maximal length without damaging the poplar leaf surface, as shown in pre-tests. Each leaf was placed on a 2% water agar (20 g micro Agar in ddH_2_O, Duchefa Biochemie, Haarlem, The Netherlands) in a Petri dish (12 × 12 cm, Greiner Bio-One International, Kremsmünster, Austria), with the petiole stuck in the agar. The Petri dishes were closed with gas permeable Parafilm® M (Bemis, Neenah, USA) and incubated in a climate cabinet (Percival Scientific, Perry, USA) at 21 °C, 60% relative air humidity, and a 16/8 h day/night rhythm. The development of lesions was inspected daily. Necrotic tissue was cut with a scalpel after 16 days and placed on PDA-S-medium (Potato-Dextrose-Agar [Carl Roth, Karlsruhe, Germany] with streptomycin [50 µg/mL, Duchefa], pH 5.5). The necrotic leaf tissue samples were incubated on PDA-S for three days in a climate cabinet as described above. Outgrowing fungal mycelia were transferred to new PDA-S plates. This step was repeated three times. Each time, the newly formed mycelia were optically differentiated according to Smith et al. [25] by color, the texture of the colony surface, and characteristics of hyphae and placed on individual plates. Based on these criteria, four optically different fungal morphotypes were differentiated. For optical identification and analysis of growth patterns, the cultures were observed under a binocular stereomicroscope (M205 FA, Leica Camera Deutschland GmbH, Wetzlar, Germany). Squeeze preparations of mycelia were generated according to the CBS Handbook of mycology [26]. Hyphal growth and morphology of conidia were observed with an inverse light microscope (Axio Observer Z1, Carl Zeiss, Oberkochen, Germany). One morphotype showed fast growth, a flat, powdery and white mycelium without aerial hyphae and branched, sporadically septated, formed hyaline hyphae, thus, fulfilling the description of *B. cinerea* [27].

The selected morphotype was used for molecular identification as follows: The genomic DNA was extracted from pinhead-sized mycelium samples with the innuPREP DNA kit (Analytik Jena, Jena, Germany) according to the suppliers’ instructions. The extracted genomic DNA was used as the template for a polymerase chain reaction (PCR), using the ITS primer pair ITS1-F *forward* (3 ‘-CTTGGTCATTTAGAGGAAGTAA-5’) and ITS4 *reverse* (5′-TCCTCCGCTTATTGATATGC-3′) [28, 29]. Following the Taq DNA polymerase protocol (Thermo Fisher Scientific, Waltham, USA), 2 µL genomic DNA in 20 µL reaction volume was used for PCR. PCR products and a technical replicate of a PCR product from a previous extraction using the same protocol as with the samples (positive control), and a ddH_2_O sample (negative control), were run on a 1.2% agarose gel containing 0.003% ethidium bromide (Carl Roth). After the run, the product lengths of the PCR products were evaluated under UV light (Typhoon FLA 9500, GE Healthcare, Chicago, USA). Signals at a size of approx. 280 base pairs indicated successful PCR. Samples were purified with the innuPREP PCRpure kit (Analytik Jena) according to the manufacturer´s instructions. Twelve µL purified PCR product was mixed with 3 µL of the primer and sent for Sanger sequencing to a company (Microsynth Seqlab, Göttingen, Germany). The resulting forward and reverse sequences were combined with the Gap4 Staden package [30]; https://sourceforge.net/projects/staden/). The resulting ITS sequences were compared with the NCBI (National Center for Biotechnology Information, Rockville Pike, USA) database using the nucleotide BLAST tool (https://blast.ncbi.nlm.nih.gov/Blast.cgi) and identified as *B. cinerea* strain CBS 261.71.

The ITS sequences obtained from two independently sequenced samples, one after isolation, and one before starting the infection experiments, were deposited in the NCBI Genbank under accession numbers ON740896 and ON740897. The *B. cinerea* cultivar isolated in this study from poplar leaves was deposited in the public collection of the German Collection of Microorganisms and Cell Cultures (DSMZ, Braunschweig, Germany) under the collection number: DSM 114,993.

### Cultivation of Botrytis cinerea

The identified fungal species *B. cinerea* was cultivated on PDA (Carl Roth) with 2% micro agar (Duchefa). Stock cultures of fungal mycelia were transferred to fresh PDA plates bi-weekly. To collect fungal mycelium, *B. cinerea* was grown for 7 days at 23 °C in darkness. To obtain spores, the fungus was grown in darkness at 28 °C for eight days [31]. For the harvest of spores, 1.5 mL 10% sterile glycerol was pipetted on the culture plate, and the spores were gently scraped with a sterile plate spreader (TH Geyer, Höxter, Germany). Spores in glycerol were stored at − 80 °C.

Liquid cultures of *B. cinerea* were produced by inoculating 100 mL Potato-Dextrose-Broth (Carl Roth) in 500 mL Erlenmeyer flasks with five 6 mm fungal agar plugs punched out with a cork borer from a seven-day-old PDA plate with *B. cinerea* mycelium. The cultures were grown for 7 days on a shaker at 120 rpm at 23 °C in darkness.

### Plug infection protocol

**Fig. 1:**
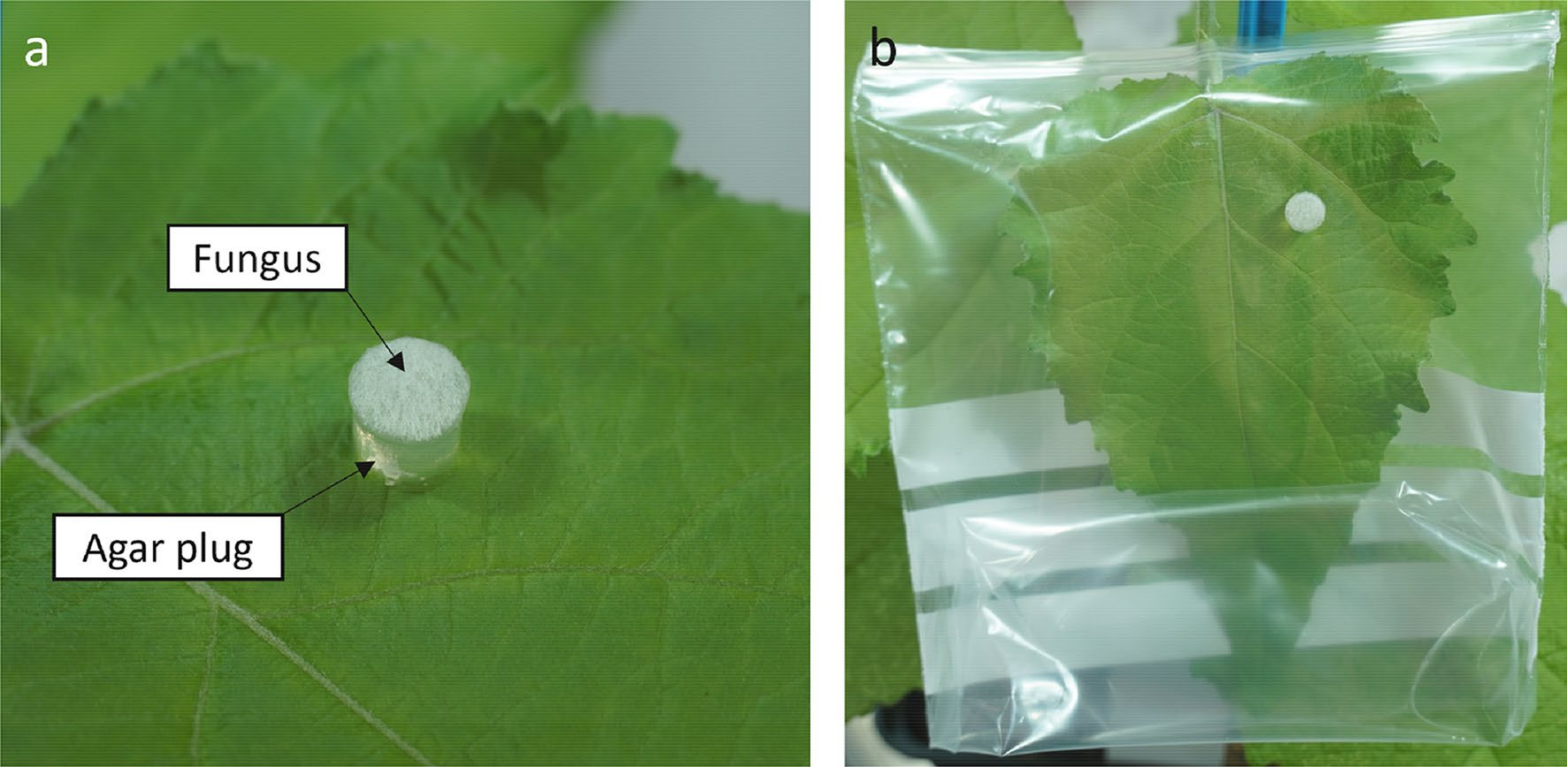
*Botrytis cinerea* infection-PDA plug on the 4th poplar leaf. From a seven-day-old *B. cinerea* culture on a Potato-Dextrose-Agar (PDA) plate, a *B. cinerea*-inoculated PDA plug was punched out with a 6 mm cork borer. **a** The *B. cinerea*-inoculated PDA plug was transferred to the 4th fully expanded *Populus* x *canescens* leaf and placed in between major veins with a needle. **b** Thereafter, the leaf was sealed with a plastic bag

PDA plugs with fungus attached were punched with a 6 mm (diameter) cork borer (Carl Roth) from the actively growing edge of a seven-day-old *B. cinerea* culture on PDA (Carl Roth). One plug was transferred with a needle on the adaxial side of an intact leaf attached to a poplar plant (Fig. 1). Generally, we used the 4^th^ fully expanded poplar leaf from the top of the shoot for the experiments. When plants were grown under non-sterile conditions, a brief surface sterilization of the leaf prior to inoculation is recommend. We wiped the upper leaf surface three times for 5 s with a 70% ethanol-soaked paper tissue interference with other potential leaf-colonizing microbes (Additional file 1: Fig. S1). Then, the plug was placed between major veins with the mycelium faced upwards (Fig. 1a). The fungus grew through the agar plug to reach the leaf surface. A PDA plug without fungus from a non-inoculated PDA plate was used for mock infection of control leaves. The growth conditions were the same as mentioned above. After inoculation, the plants were cultivated for up to four days under ambient light with 16 h of supplemental illumination of 150 µE m^−2^ s^−1^ photosynthetically active radiation. The temperature ranged from 15 °C to 30 °C, and air humidity was between 53 and 82%. The plants were watered regularly with tap water. Infected and non-infected leaves were harvested at different days post inoculation (dpi).

**Fig. 2:**
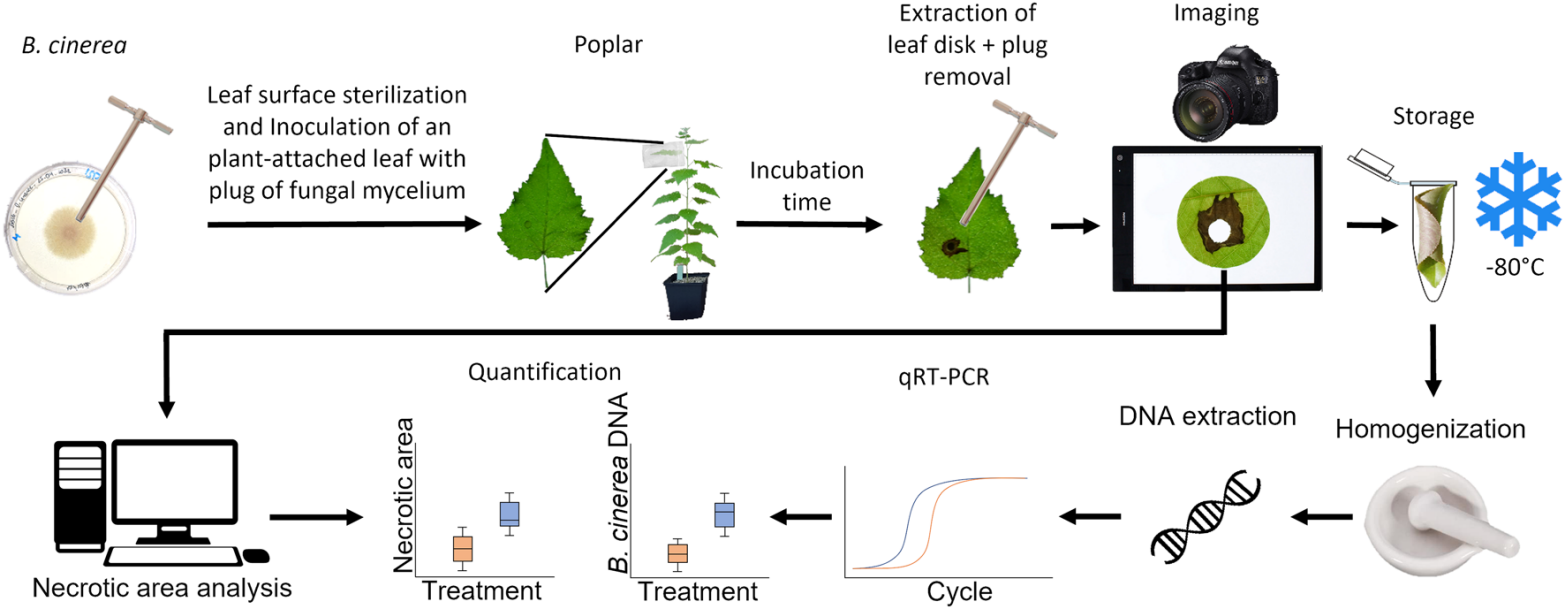
Flow chart of the poplar-*Botrytis cinerea* pathoassay inoculation, harvest process, and quantification

To inoculate leaves from the outdoor poplars, fully expanded leaves attached to twigs were chosen. Inoculated leaves were placed in a low-density polyethylene zipper bag (Carl Roth). The lower end of the bag was pushed slightly inside the bag to create an air space to ensure that the plug did not stick to the bag (Fig. 1b). The plastic above the zipper was cut-off to ensure sufficient humidity and spread of fungal material while being able to place the zipper as near as possible to the stem (Fig. 1b). A flow chart of the infection method and tissue harvest is shown in Fig. 2.

The 4th leaf of *Populus* x *canescens* was sterilized by wiping the upper leaf surface three times for five seconds with a 70% ethanol-soaked paper tissue. Plugs were punched out at the actively growing edge of a 7-day-old *B. cinerea* culture. One plug was placed with the mycelium upwards on the *Populus* x *canescens* leaf. The leaf was sealed in a plastic bag. Leaves were harvested after different incubation times and digital pictures were taken. A leaf disk (diameter: 27 mm) around the centered necrosis was plunged out, and the area of the initial plug position was removed with the 6 mm cork borer before the sample was snap-frozen in liquid nitrogen and stored at − 80 °C. The total DNA was extracted from the leaf disks. Fungal DNA was measured via qRT-PCR. Ct-values were converted into the amount of DNA. The necrotic area was determined from the photos of the leaf disks using the R package pliman (Olivoto, 2022).

### Spore infection protocol

To test the ability of the spores to germinate, harvested spores were allowed to germinate for 24 h in darkness at 23 °C. Twenty microliters of germinated spores on a glass slide were mounted with glycerol and observed with an Axio Observer Z1 microscope (Carl Zeiss). Photos were taken with an AxioCam MRc (Carl Zeiss).

For inoculation, spores were diluted to a concentration of 1 × 10^6^ [31] in tap water, and a volume of 20 µL was pipetted onto the fourth, fifth, and sixth fully developed leaf of a –greenhouse-cultivated *P*. x *canescens* plant. The inoculated leaves were individually enclosed in plastic bags to maintain high air humidity and incubated as described above. The leaves were monitored regularly.

### Sampling of leaf disks

Inoculated leaves were cut from the stem with a blade after one to four days of infection. If not stated otherwise, the infection plug was punched out with a 6 mm cork borer and discarded. A leaf disk (27 mm diameter) was punched out around the 6 mm-wide hole. For further analyses, these leaf disks were used. The advantage of this approach is that we could use normalized leaf areas for image processing and fungal DNA quantification. Leaf disks for DNA quantification were frozen in liquid nitrogen and stored at − 80 °C (Fig. 2).

### Determination of lesion area by image analysis

All photographs of leaves and leaf disks were taken with a Sony Alpha 6400 camera with a Sony SEL 18-135 mm f3.5–5.6 lens (Sony, Tokyo, Japan) and the following settings: 1/60 s exposure time, aperture F5.6, iso 200, 55 mm zoom, and manual focus. The samples were placed on an LED light table (Shenzhen Huion Animation Technology, Shenzhen, China) with maximum light intensity. A scale bar was included in each photograph, and no additional light source was used to avoid reflections.

The digital pictures of the leaf disks were used to determine the necrotic area. Infected areas were defined manually using a freely available imaging program (GIMP v2.10.32, https://www.gimp.org/, The GIMP Development Team, 2022). We distinguished green, healthy leaf tissue and brownish, pathogen-affected tissue using the foreground color selection tool and colored the necrotic area in bright red. The processed images were stored as png files and opened in ImageJ (Schindelin et al., 2012). The scale on each image was used to define the cm-to-pixel ratio, and then the necrotic area was determined using the “ROI Manager” tool. The total leaf area of interest (TLAI) was defined as the total leaf area of the disk minus area of the punched hole. TLAI and infected area were used to calculate the relative infected area.

R [32] and Rstudio [33] with the pliman package v 1.1.0 [34] were used for the automated analysis of symptomatic areas. For each experiment, corresponding references (i.e., images of healthy tissue, necrosis and background, img_healthy, img_symptoms, and img_background) were recorded and used in GIMP v2.10.32 [35]. Batches of pictures were analyzed using the pattern function of “pliman”. The function to fill holes (fill_hull = FALSE) was disabled to achieve correct recognition of the punched hole. Processed pictures were saved (save_image = TRUE) to check for correct processing by inspection of the white lines the R package draws around the recognized necrotic area. After processing, percent values of necrotic and healthy areas were saved in.csv format. The script used here was deposited at Github (https://github.com/) under: https://github.com/StevenDreischoff/Fast-and-easy-infection-assay-for-necrotizing-pathogen-Botrytis-cinerea-on-Poplar

### Calibration and Quantification of Fungal DNA by qRT-PCR

Frozen leaf disks (TLAI) were milled twice in a ball mill (MM200, Retsch, Haan, Germany) equipped with two 3 mm and one 4 mm steel balls for 90 s at 30 Hz. Total DNA was extracted from frozen leaf powder using the innuPREP PlantDNA Kit (Analytik Jena) with an extended lysis time of 1 h. Afterward, the DNA concentration and purity were determined in 1 µL of extract in a NanoDrop One (Thermo Fisher Scientific) spectrophotometer. The DNA concentrations of the leaf extracts were adjusted to 5 ng/µL.

We employed the *B. cinerea*-specific primers Bc3F (5’-GCTGTAATTTCAATGTGCAGAATCC-3’) and Bc3R (5’-GGAGCAACAATTAATCGCATTTC-3’) reported by Suarez et al. [36] and Diguta et al. [23] to quantify fungal DNA in the leaf extracts. The primers target the ribosomal region between the 28S and 18S genes (intergenic spacer). The quantitative real-time PCRs (qRT-PCR) reaction mixture contained 10 µL innuMIX qPCR DSGreen Standard master mix (Analytik Jena), 5 µL DNA (concentration 5 ng/µL, equaling 25 ng total DNA), 4 µL ddH_2_O and 1 µL primer mix of forward and reverse primer (10 µM each). The qRT-PCRs reactions were carried out with a qTower3G (Analytik Jena) at the following conditions: initial denaturation at 95 °C for 2 min, 40 cycles of 1 denaturation at 95 °C for 20 s, 2 annealing at 58 °C for 20 s and 3 elongation at 72 °C for 20 s. The qRT-PCR ended with a melting curve, starting from 60 °C to 95 °C in 15 s with an increase of 5 °C per s. Three technical replicates were analyzed per biological sample. Determination of Ct-values was performed with the qPCRsoft-Software v4 (Analytik Jena).

To calibrate the fungal Ct values, we produced pure *B. cinerea* DNA. For this purpose, mycelium was harvested from a liquid culture after one week of growth. The whole mycelium from one culture was lyophilized (Gamma 2–16 LSCplus, Martin Christ Gefriertrocknungsanlagen GmbH, Osterode am Harz, Germany). A hundred mg of freeze-dried mycelium was rehydrated with 150 µL ddH_2_O and immediately used for DNA extraction with the DNeasy PowerSoil Pro Kit (Qiagen, Hilden, Germany) following the instructions of the manufacturer. The absorbance ratios at 260/280 nm and 260/230 nm were checked with a NanoDrop One spectrophotometer (Thermo Fisher Scientific). Thereafter, the DNA was further purified with the Dneasy PowerClean Pro Cleanup Kit (Qiagen) according to the manufacturer’s protocol. The resulting DNA concentration was determined using a Qubit dsDNA HS assay Kit in a Qubit 3.0 Fluorometer (Thermo Fisher Scientific). It should be noted that the extraction procedure was successful with the Qiagen soil but not with the Analytik Jena plant kit.

The purified DNA from the fungal mycelium was used to produce a dilution series from 14.8 ng µL^−1^ to 0.00001 ng µL^−1^. The samples were used for qRT-PCR according to the following conditions: the qRT-PCR reaction mixture contained 10 µL innuMIX qPCR DSGreen Standard master mix (Analytik Jena), 5 µL DNA, 4 µL ddH_2_O and 1 µL primer mix (Bc3F and Bc3R) of forward and reverse primer (10 µM). The qRT-PCRs were carried out with a qTower3G (Analytik Jena) and the following conditions: initial denaturation at 95 °C for 2 min, 40 cycles of 1 denaturation at 95 °C for 20 s, 2 annealing at 58 °C for 20 s and 3 elongation at 72 °C for 20 s. The qRT-PCR ended with a melting curve, starting from 60 °C to 95 °C in 15 s with an increase of 5 °C per s. Three technical replicates were analyzed per biological sample. Determination of Ct-values was performed with the qPCRsoft-Software v4 (Analytik Jena).

**Fig. 3:**
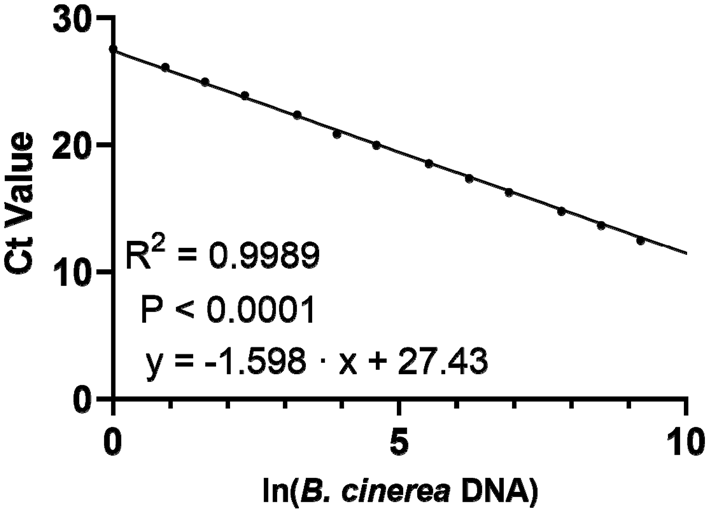
Calibration curve for the amount of fungal DNA (pg) to Ct values

The Ct values were regressed against the amount of DNA after ln (natural logarithm) transformation of the DNA values. A correlation test was performed using R [32] and Rstudio [33]. Data were plotted, and linear regression was performed (Fig. 3). We obtained a linear relationship (Ct = -1.598 ln(DNA) + 27.43, R^2^ = 0.9989, P < 0.0001). The equation was used to convert Ct values into corresponding amounts of fungal DNA (in pg) with the following Eq. 1:

1 $${DNA}_{(pg)}= {e}^{(\frac{27.43- Ct value}{1.598})}$$

For all experiments, Ct values were converted into amounts of fungal DNA in pg. These amounts corresponded to the amount of fungal DNA in 25 ng of total (plant + fungal) DNA.

Fungal DNA was extracted from freeze-dried *Botrytis cinerea* mycelium grown in liquid culture. The purified DNA was serially diluted. qRT-PCR was performed using *B. cinerea*-specific primers. For every sample, three technical replicates were analyzed. Mean Ct values of the technical replicates were correlated to the amount of DNA in the PCR reaction. DNA data were ln transformed since the Ct values increase exponentially.

### Scanning electron microscopy (SEM) of fungal mycelia on leaf surfaces

Fresh leaf samples (approximately 1 cm × 1 cm) were cut at different positions and time points after inoculation from infected and control leaves with a double-edge razor blade (Wilkinson Sword, Solingen, Germany). Each sample was fixed with conductive double-sided adhesive carbon tabs (Plano, Wetzlar, Germany) on a Standard SEM Pin Stub Mount (diameter 12.7 mm, Phenom-World, Eindhoven, Netherlands). Immediately afterwards, the samples were covered by a 10 nm gold layer (Plano) using the Q150R S/E/ES plus sputter coater (Quorum Technologies, Lewes, United Kingdom). After sputter coating, the edges of the samples were sealed airtight with conductive carbon cement (Plano). The samples were stored in a dust-free SEM sample box for a maximum of two days before the measurements were conducted. SEM images were taken on a Phenom ProX (G5) desktop SEM (Thermo Fisher Scientific). Image acquisition was performed in Back-scattered electron mode (BSE mode) at a voltage of 15 kV and a resolution of 1024 pixels, and magnifications of 350x, 1000x, and 4000x.

### Chlorophyll fluorescence imaging

Chlorophyll fluorescence of sections of whole leaves, including inoculated areas, was measured with an IMAGING-PAM (Heinz Walz, Effeltrich, Germany). The measurements were conducted at an intensity of 0.5 µmol m^−2^ s^−1^ PAR, at a modulation frequency of 8 Hz, and at a saturation pulse intensity of 4000 µmol m^−2^ s^−1^ PAR. The emission of blue light was measured at 450 nm and of red light at 650 nm. According to Sekulska-Nalewajko et al. [37], the photosynthetic efficiency of dark-adapted leaves (Fv/Fm) and the quantum yield on non-regulated PSII energy dissipation (Y(NO)) data were used to analyze the impact of *B. cinerea* infection on leaf photosynthesis.

### Cross-sections and histochemistry of infected and uninfected leaves

Fresh leaf samples of 1 cm x 1 cm were cut with a razor blade (Wilkinson Sword) from different parts of mock and *B. cinerea*-infected leaves. The samples were transferred individually in 2 cm x 2 cm wells of a sixteen-well plate (Greiner Bio-One International). The wells were then filled with liquid 5% agarose (Duchefa Biochemie), closed with a lid, and solidified at 4 °C overnight. During initial solidification, the samples were pushed down several times with forceps to prevent floating.

The agarose blocks containing the leaf samples were removed from the well plate, orientated for cross-sectioning, and trimmed with a razor blade (Wilkinson Sword). The vibratome VT1200 (Leica Microsystems, Wetzlar, Germany) was used to produce cross sections of 30 µm thickness with a cutting speed of 1 mm sec^−1^ and an amplitude of 0.5 mm. For this purpose, the vibratome was equipped with a double-edge bendable razor blade (Wilkinson Sword). The cross-sections were then submerged in a solution containing 10 µg mL^−1^ Wheat Germ Agglutinin Alexa Fluor 488 conjugate (Thermo Fisher Scientific) and propidium iodide 10 µg mL^−1^ (Thermo Fisher Scientific) in phosphate-buffered saline solution (NaCl 137 mmol L^−1^, KCl 2.7 mmol L^−1^, Na_2_HPO_4_ 10 mmol L^−1^, KH_2_PO_4_ 1.8 mmol L^−1^) for 15 min. After staining, the cross-sections were washed once with phosphate-buffered saline solution and then transferred to specimen slides, where they were mounted with ROTIMount FluorCare (Carl Roth), a medium for fluorescence microscopy. The sections were viewed at 200 × and 400 × magnification with a Zeiss Axio Observer Z1 microscope (Carl Zeiss), using filters with the wavelengths for excitation at 493 nm and emission at 520 nm for WGA Alexa Fluor 488 conjugate and with excitation at 538 nm and emission at 617 nm for propidium iodide. Photos were taken with an AxioCam MRc (Carl Zeiss).

### Methyl jasmonate pretreatment

Ten-week-old greenhouse-grown *P.* x *canescens* plants were inserted in a large autoclave bag (Sarstedt, Nümbrecht, Germany) with open ends at the top and the bottom. This setup prevented cross-contamination during spraying. The plants were sprayed with 200 µM methyl jasmonate (Sigma-Aldrich, St. Louis, USA) in ddH_2_O or with ddH_2_O (control) until runoff (approx. 25 mL) using a 250 mL spray bottle suitable for overhead spraying (Carl Roth). The bags were closed, and the control and MeJA-treated plants were transferred to different greenhouse cells. The bags were removed after four hours, and the plants were incubated for another four hours before inoculation and incubation *with B. cinerea*, as described above.

### Statistical analysis

Data are shown as box plots with points representing one biological replicate unless indicated otherwise. The number of biological replicates is indicated in the tables and figure legends. To compare means, the normal distribution of the data sets was tested by visual inspection of residuals. If data were not normally distributed, they were log-transformed to achieve normal distribution. If data in percent included 0 or 1, they were transformed according to Smithson & Verkuilen [38]. Statistical tests were conducted with R [32] and Rstudio [33], using linear generalized mixed models, beta regression [39], or Tukey’s post hoc-test in the packages”multcomp” [40] and”car” [41]. Differences between treatments were considered significant when the values of the post hoc tests were P < 0.05. Correlations were tested using the build-in cor.test() function of R.

## Results

### *Botrytis cinerea* plug-inoculation results in spreading necrosis on the poplar leaves

**Fig. 4:**
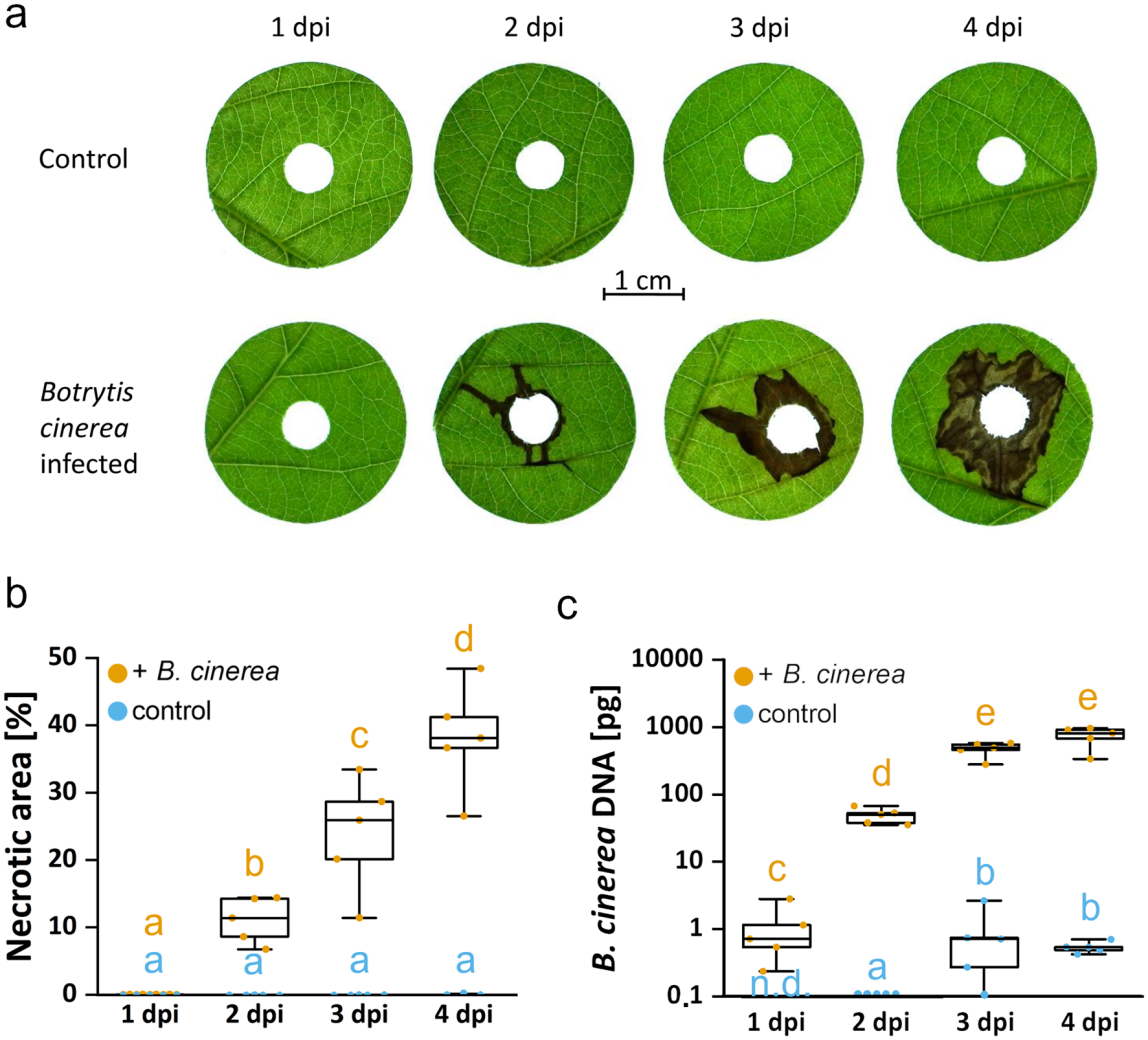
Time course of disease symptoms **a**, **b** and amount of fungal DNA **c** in poplar leaves in response to *Botrytis cinerea*

Leaves of *P*. x *canescens* did not show necrotic symptoms one day after *B. cinerea* inoculation. After 2 dpi, initial dark brownish necroses were observed, which grew in size at 3 and 4 dpi (Fig. 4a). Analyses of the necrotic areas showed significant increases from 2 to 4 dpi (Fig. 4b).

To be able to measure the *B. cinerea* DNA in the leaf disks, *B. cinerea* DNA extracted from pure culture was correlated to Ct values (Fig. 3). We quantified the amount of fungal DNA in infected leaves and mock-inoculated controls with *B. cinerea-*specific primers. *B. cinerea* DNA was detectable at all tested time points and increased significantly until 3 dpi (Fig. 4c). At 4 dpi, no further increase was detected (Fig. 4c). Mock-inoculated leaves also showed minor increases in *B. cinerea* DNA (Fig. 4c). This observation suggests that the fungus was ubiquitously present in leaves of greenhouse-grown poplars and that its growth was stimulated by the application of the uninfected plug during the incubation period.

Leaves of *Populus* x *canescens* were inoculated with a seven-day-old *B. cinerea* plug punched out of Potato-Dextrose-Agar plate or were mock inoculated with a plug from a sterile Potato-Dextrose-Agar plate. Leaves were harvested after one, two, three-, and four days post inoculation (dpi) as described in Figs. 1 and 2. a) Visual symptom development, b) increase of the necrotic area, c) and amount of *B. cinerea* DNA within the time course of 4 days. Quantification of necroses was conducted with the pliman package and expressed as percentage of the necrotic area of the leaf disk area (5.44 cm^2^). Fungal DNA was extracted from leaf disks (5.44 cm^2^), analyzed with *B. cinerea*-specific primers, and converted to the amount of *B. cinerea* DNA using the calibration in Fig. 3. Data are shown as min–max boxplots with the whiskers spanning across the range of the data points. Each point represents one biological replicate (n = 5). Each biological replicate was determined as the mean of three technical replicates. Different letters above the boxplots indicate significant differences at P < 0.05 calculated by ANOVA and Tukey post hoc test. n.d. = not detectable.

**Fig. 5:**
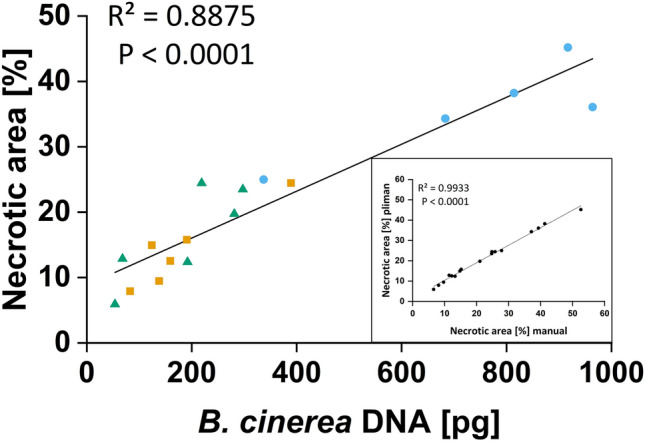
Relationship of necrotic leaf area and the amount of *Botrytis cinerea* DNA

The infection of *P*. x *canescens* leaves by *B. cinerea* was independently repeated three times to evaluate the reproducibility of the pathosystem. Necrosis developed on the *B. cinerea* inoculated leaves, but the symptomatic areas differed among independent experiments (Fig. 5). This result was probably due to differences in the ambient, semi-controlled glasshouse conditions during the inoculation period or different effects of the conditions on the poplar plants. The necrotic areas were highly correlated with the amount of detected *B. cinerea* DNA (Fig. 5), indicating the proper functioning of the pathosystem also under changing environmental conditions.

In three independent experiments, the 4th fully developed leaf of a *Populus* x *canescens* plant was inoculated with a *B. cinerea-*plug from a seven-day-old Potato-Dextrose-Agar plate and harvested after four days of inoculation as described in Figs. 1 and 2. The necrotic area was determined from images using both the pliman package and manually with ImageJ. Necrotic areas were expressed as percentages of the area of the total leaf disk (5.44 cm^2^). Different amounts of fungal DNA from different experiments (blue circles, green triangles, orange squares) showed a significant correlation with the necrotic leaf area. Both methods (pliman and manual necrotic area determination) show a highly significant correlation (inset in Fig. 5). Each symbol represents one biological replicate. Each biological replicate was determined as the mean of three technical replicates.

In the initial experiments, we determined the necrotic area on poplar leaves manually, as commonly done in other studies, e.g., [42, 43]. This procedure was very labor-intensive. The recently published R package pliman [34] was used to automatize and speed up the analysis. To test whether pliman delivers reliable results, the necrotic areas found by manual and pliman analyses were compared. Both methods produced similar results and were highly significantly correlated (Inlet Fig. 5). Complete setup and image analysis with pliman took about 20 min, while the manual analysis took approx. three minutes per sample. Thus, pliman outperforms the manual approach by being faster and less laborious when dealing with seven or more samples. The optimized pliman procedure started with reference images for an individual experimental run, which was used to correct for the slight reddish coloration which often appears on poplar leaves.

### Inoculation with *Botrytis cinerea* mycelium plugs results in the infection of different poplar species

**Fig. 6:**
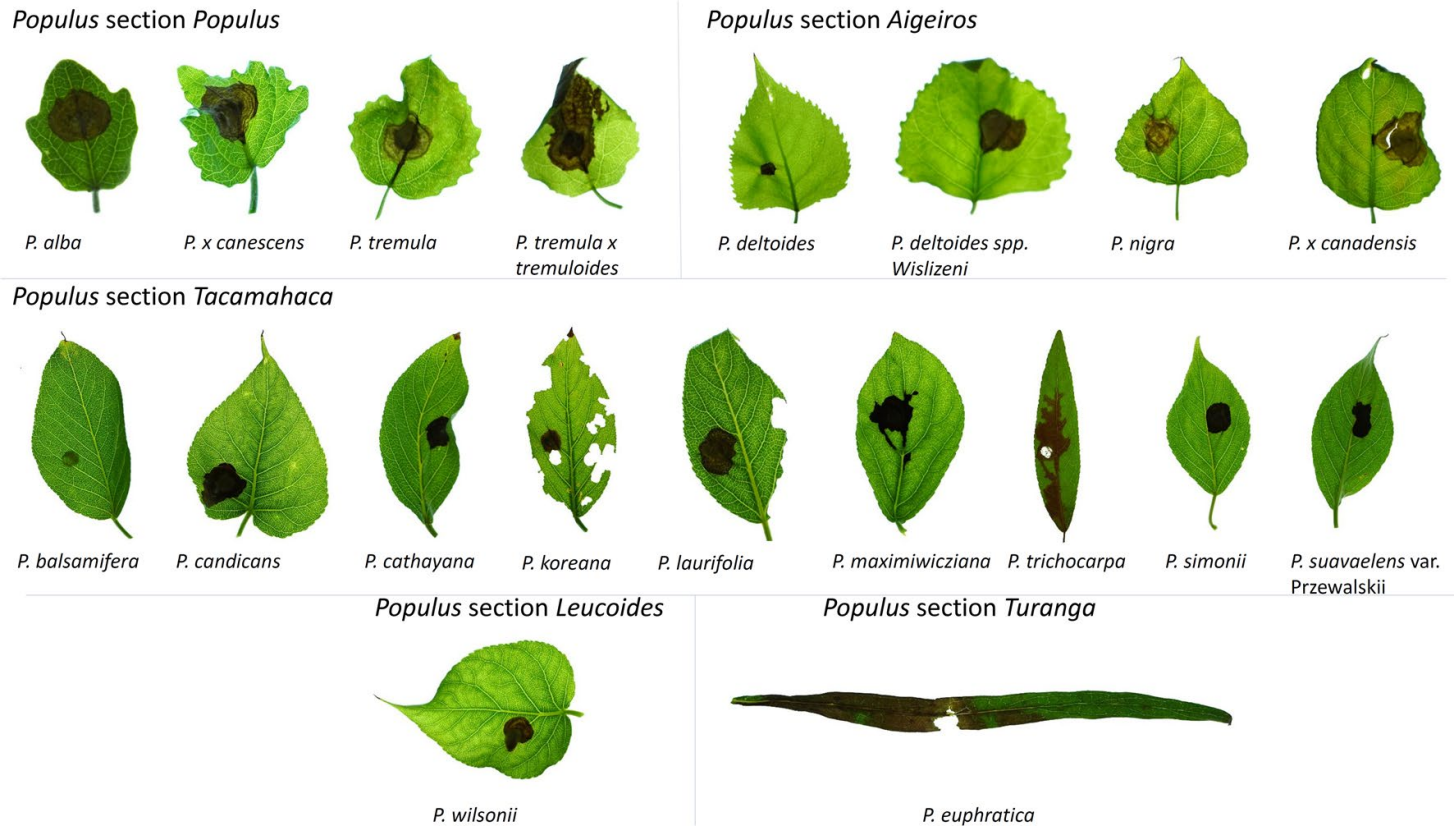
*Botrytis cinerea*-induced necroses on leaves of poplar species different sections

To test the versatility of the infection system, leaves of poplar species of different sections cultivated under controlled conditions or collected in the Forest Botanical Garden (University of Goettingen, Germany) were inoculated with *B. cinerea* plugs. All tested species from the sections *Populus, Aigeiros, Tacamahaca, Leucoides,* and *Turanga*, except *P. balsamifera*, were successfully infected and showed necroses on day four after inoculation (Fig. 6).

Different poplar species were obtained from greenhouse stocks and the Forest Botanical Garden Göttingen of the University of Goettingen (Germany). Leaves attached to twigs or whole plants were inoculated with a *B. cinerea* plug from a seven-day-old Potato-Dextrose-Agar plate and harvested four days post inoculation, as described in Figs. 1 and 2. *Populus deltoides* and *P. koreana* leaves were additionally damaged by caterpillars during the infection period. Pictures were taken of the full leaves, and brightness was enhanced by 20% for better visualization.

### Histological analyses of infected leaves underline the necrotizing lifestyle of Botrytis cinerea

**Fig. 7:**
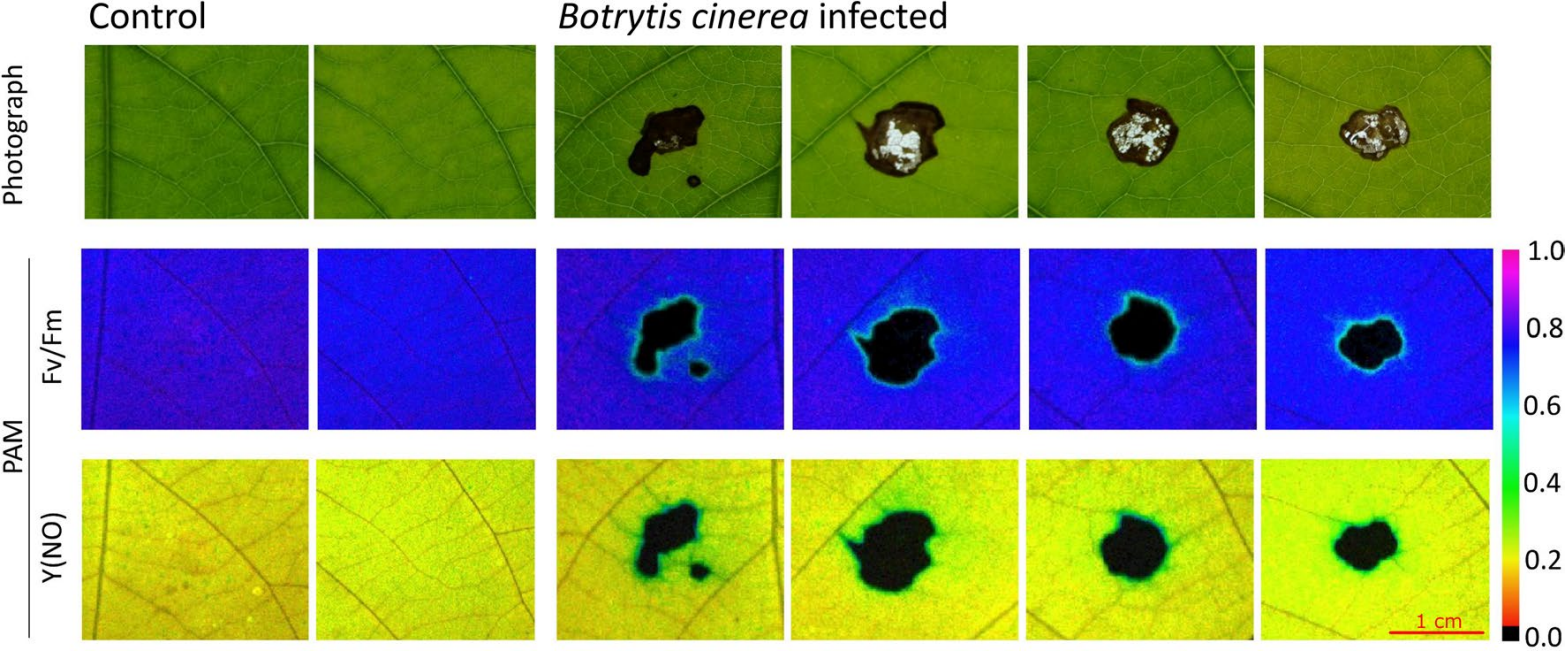
Images of control and *Botrytis cinerea* infected *Populus* × *canescens* leaves captured by light photography (top row) and pulse amplitude modulation (PAM) fluorometry (middle and bottom rows). Fv/Fm: Photosynthetic efficiency of dark-adapted leaves, Y(NO): quantum yield of non-regulated PSII energy dissipation. Leaves were inspected 4 days post inoculation

To obtain insights into the response to fungal infection, we analyzed the chlorophyll fluorescence of *B. cinerea-*infected and uninfected *P*. x *canescens* leaves. At 4 dpi, typical necroses were observed below and around the *B*. *cinerea*-PDA infection plugs but not on control leaves inoculated with pure PDA plugs (Fig. 7, upper row). The maximum quantum yield (Fv/Fm) of photosystem II of control leaves and the non-disturbed tissue of infected leaves were close to 0.8 (dark blue color Fig. 7 middle row). The infected leaves show reduced quantum yield, indicated by the light blue halo surrounding the necrotic area (black). The non-regulated energy dissipation (Y(NO)) was low (orange to yellow) in controls (Fig. 7 bottom row) and increased close to necrotic areas (dark green color in Fig. 7 bottom row). While photosynthetic damage (Fv/Fm) occurred only at the edge of the necrotic area, non-regulated energy dissipation was already observed in a larger area of the infected leaves surrounding the necrotic tissue (Fig. 7, middle and bottom row).

**Fig. 8:**
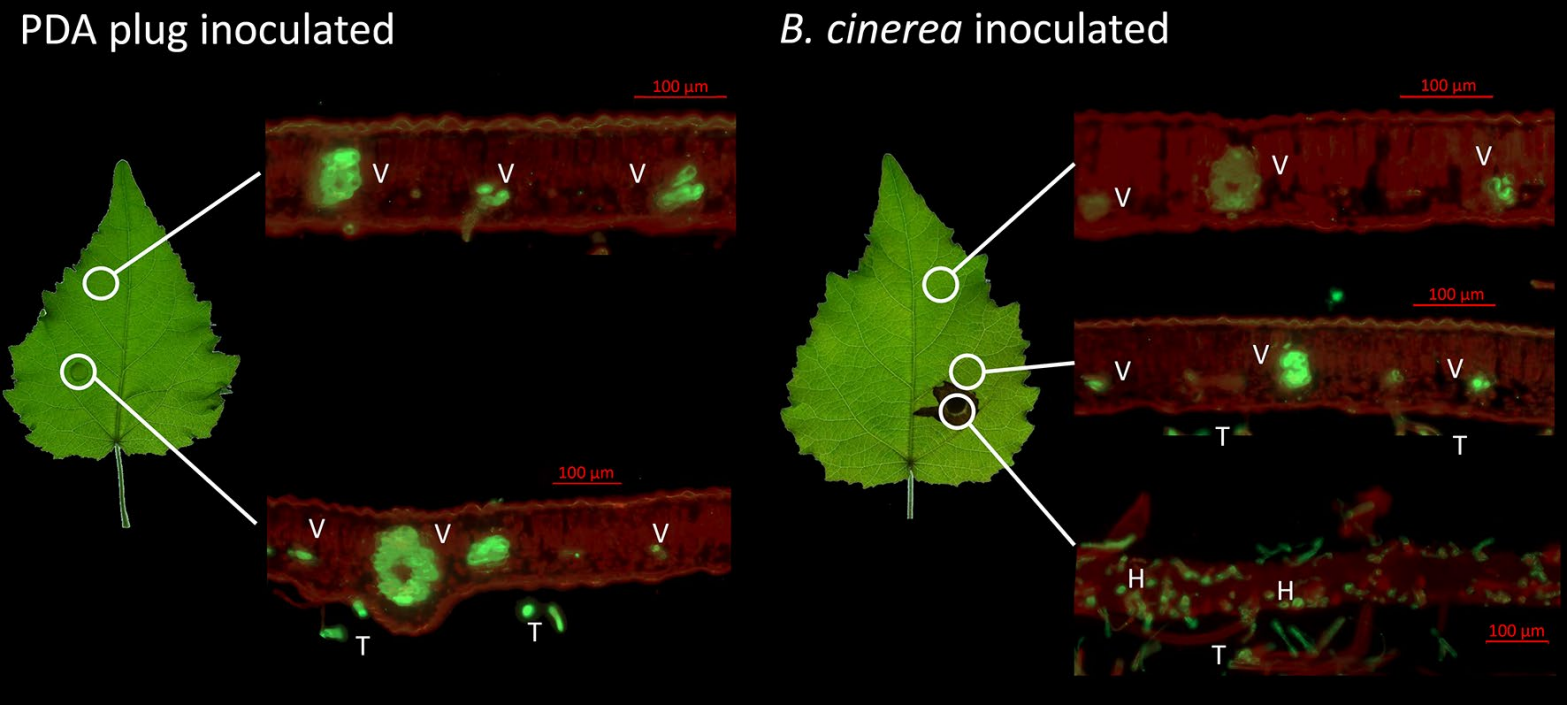
Fluorescence of leaf cross sections at different positions of mock and *Botrytis cinerea* inoculated *Populus* x *canescens* leaves

To inspect the presence of *B. cinerea* hyphae outside the necrotic area, leaf cross sections of mock- and *B. cinerea*-inoculated leaves were stained to visualize fungal hyphae and plant cell walls (Fig. 8). Leaf structures were disintegrated in the necrotic areas, and hyphae could be observed all over the broken tissue. No hyphae could be identified in the tissue near the necrosis, away from the necrosis, or mock-inoculated leaves (Fig. 8).

The 4th fully developed leaf of *P.* x *canescens* was inoculated with a *B. cinerea* plug from a seven-day-old Potato-Dextrose-Agar plate or mock inoculated with a plug from a sterile Potato-Dextrose-Agar plate and harvested four days post-inoculation. Leaf tissues (1 cm × 1 cm) from the positions indicated in the figure were embedded in agarose, trimmed after solidification, and sectioned with a vibratome (thickness 30 µm). The sections were stained with 10 µg mL^−1^ propidium iodide to visualize cell walls (red) and 10 µg mL^−1^ Wheat Germ Agglutinin Alexa Fluor 488 conjugate to visualize the fungus (green). Photos were taken under a fluorescence microscope. V = vascular bundle, T = trichome, H = hyphae.

**Fig. 9:**
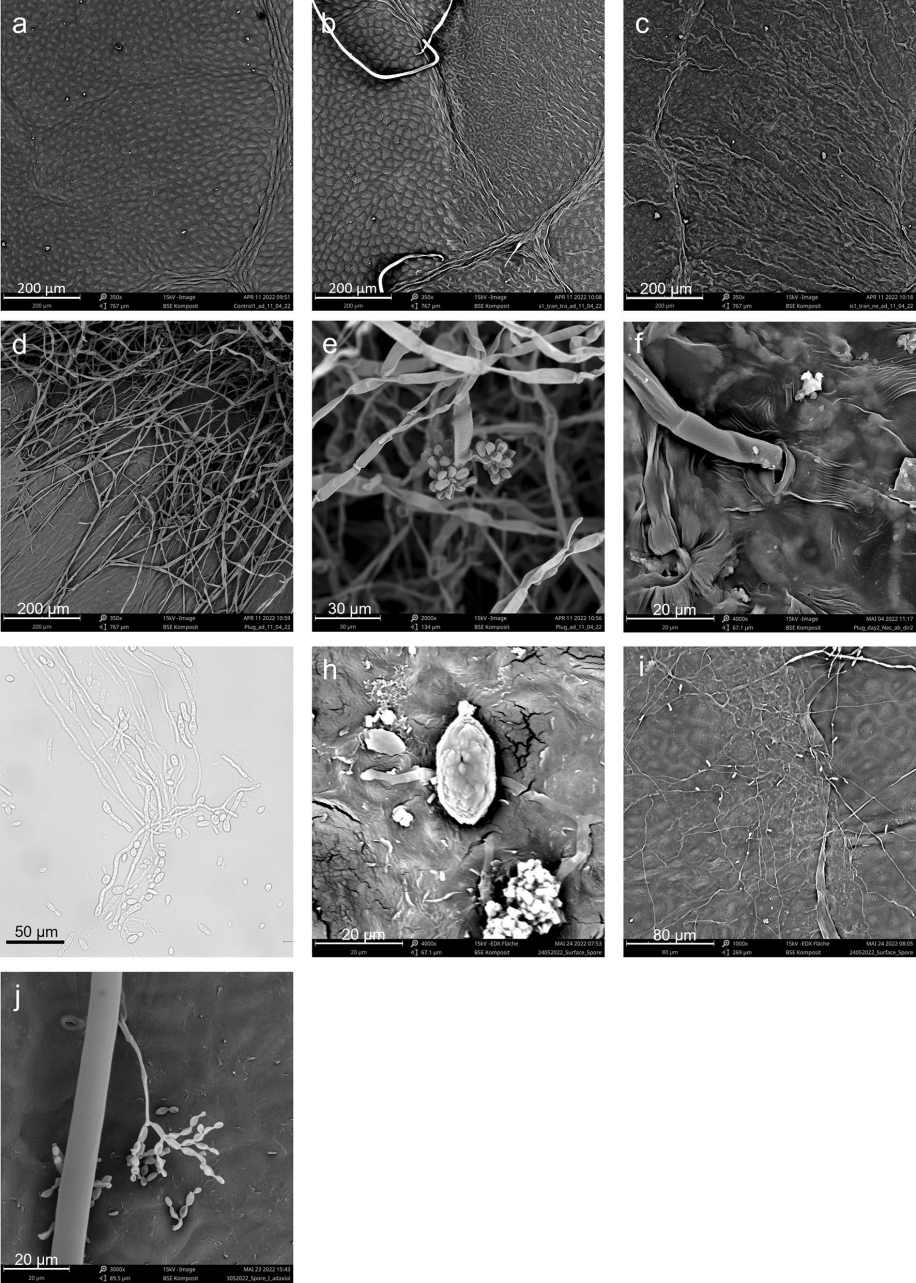
Scanning electron microscopy of *Botrytis cinerea* structures on inoculated *Populus* x *canescens* leaves **a**–**f**, **h**–**j** and light microscopy of *B. cinerea* in liquid culture **g**. **a** Sterile PDA-plug inoculated leaf. **b** *B. cinerea* inoculated leaf. Edge of the necrotic (right) and healthy (left) area after four days. **c**) Collapsed cells of a *B. cinerea* inoculated leaf at 4 dpi (days post inoculation). **d** Mycelium growing from the plug to the leaf at 2 dpi. e) Spore on the abaxial side at 3 dpi. **f** Mycelium growing through a stoma at 3 dpi. g) *B. cinerea* spores germinated 24 h in ¼ strength Potato-Dextrose-Broth, **h** *B. cinerea* spores on inoculated leaf at 12 dpi, **i** germinated *B. cinerea* spores forming a hyphal network on poplar leaf at 12 dpi, **j** *B. cinerea* conidiophores at 12 dpi

In addition, scanning electron microscope pictures were taken from inoculated leaves after different time points and at different positions on the leaves. Non-necrotic areas of infected leaves did not show any differences in the epidermal cell patterns compared to healthy leaves (Fig. 9a, b). In necrotic areas of the adaxial side of the leaf, the epidermal cells appeared to be sunken and less turgid, thus, being distinguishable from healthy cells in the direct neighborhood (Fig. 9b). Necrotic tissue showed no clear vital cell patterns, only epidermal ridges (Fig. 9c). At the beginning, mycelium grew from the plug toward the leaves, then, forming a network on the leaf surface, similarly as it does on culture medium. After 2 dpi, hyphae were present on the abaxial side of the leaf directly under necrotic areas, showing fungal trespassing through the leaf (Fig. 9e). Fungal spores (Fig. 9e) and hyphae penetrating the stomatal pores were detected under the necrotic areas on the lower leaf surface (Fig. 9f).

We also tested the ability of fungal spores to induce leaf necrosis on *P. x canescens.* We inoculated the leaves with 1 × 10^6^ spores suspended in water or ¼ PDA. The treatments resulted in irreproducible infection patterns. The spores germinated on ¼ PDA (Fig. 9g), on the leaves (Fig. 9h) and formed hyphal networks (Fig. 9i) and conidia (Fig. 9j). But necrosis did not occur or was only very minor, even after long incubation periods (> 14 dpi). This result shows that the infection is hardly promoted under normal conditions, even when the leaves are kept in high humidity.

### Jasmonate-dependency of the infection

**Fig. 10:**
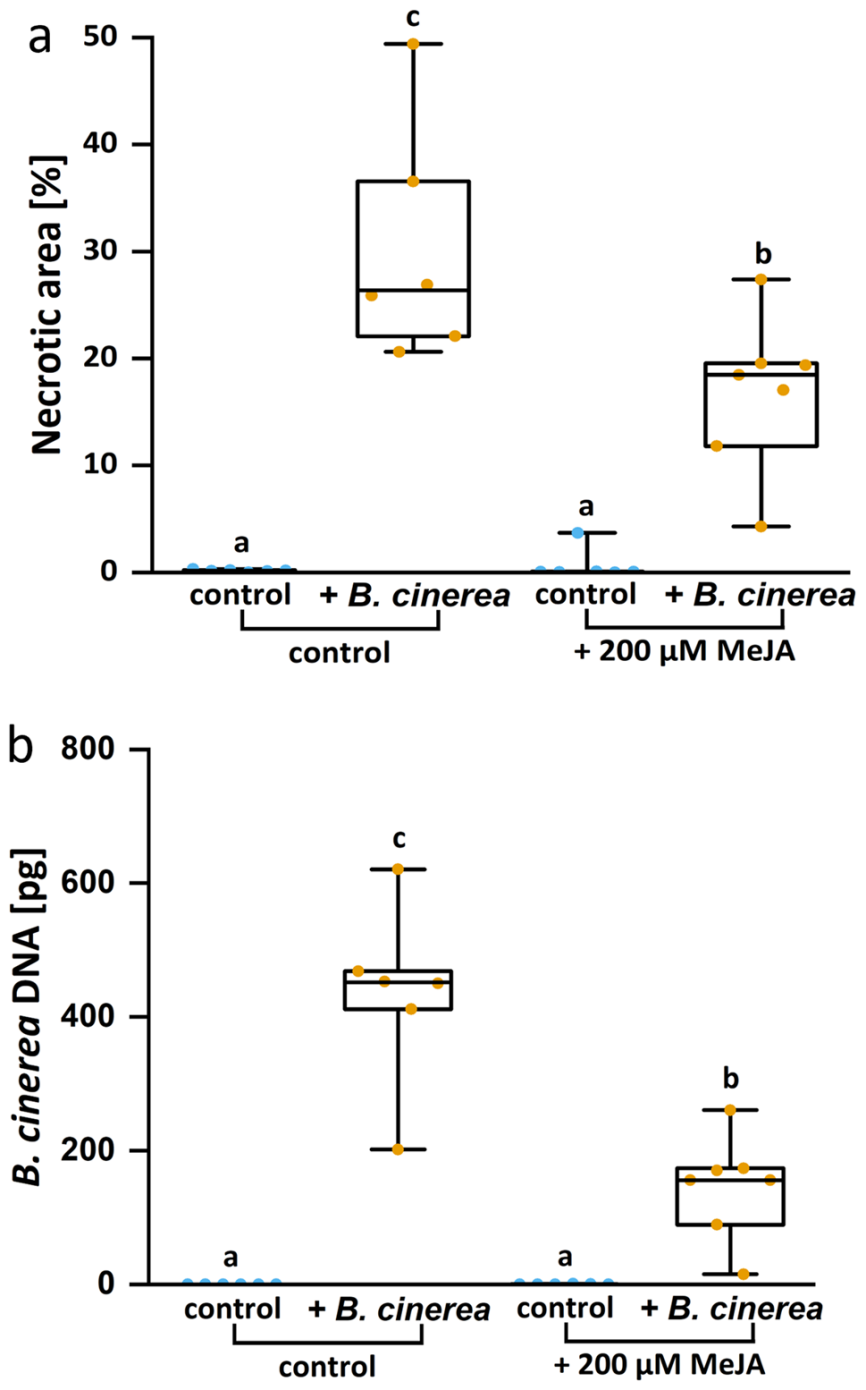
Leaf necrotic area **a** and *Botrytis cinerea* development **b** in poplar leaves after methyl jasmonate pretreatment

Jasmonate is involved in the defense against necrotizing fungi in many plant species [44]. To test whether our bioassay system is capable of capturing jasmonate-responses of poplar leaves, whole *P.* x *canescens* plants were pretreated with methyl jasmonate. After 8 h pretreatment, leaves were plug-inoculated with *B. cinerea*. Mock-inoculated leaves did not show symptoms, whereas inoculated leaves showed visible necroses after 4 dpi. Methyl jasmonate exposed plants showed significantly less necrotic area and fungal DNA than the non-induced plants (Fig. 10).

Ten-week-old greenhouse-grown *Populus* x *canescens* were sprayed with 200 µM methyl jasmonate (MeJA) in ddH_2_O or with ddH_2_O (control) until runoff (approx. 25 mL). Eight hours after pretreatment, the 4^th^ fully developed leaf was inoculated with a *B. cinerea* plug from a seven-day-old Potato-Dextrose-Agar plate and harvested four days post inoculation. The necrotic area was determined with the pliman method and expressed as percentage of the total leaf disk area (5.44 cm^2^). *B. cinerea* growth was quantified by qRT PCR and calibrated with the data in Fig. 3. The figures show min–max boxplots with the whiskers spanning across the range of the data points. Each point represents one biological replicate (n = 6–7). Each biological replicate represents the mean of three technical replicates. Different letters above the box plots indicate significant differences at P < 0.05 calculated by ANOVA and Tukey post hoc test.

## Discussion

Here, we developed a simple method that leads to the infection of poplar leaves by *B. cinerea* hyphae. We show that under these conditions, a range of phylogenetically divergent poplar species [45] can be infected, whereas *B. cinerea* infections are rare when the leaves were exposed to spores. Within the first days after inoculation, the increase in necrotic area reliably reported the severity of *B. cinerea* poplar leaf infections.

The infection can be performed with basic laboratory materials. While the automated analysis of the necrotic area using pliman is an improvement to individual analyses with imaging software in terms of speed and threshold assignment, it is still facing other problems in phytopathometry, such as the need for expertise in the classification of disease symptoms [21]. The qRT-PCR-based fungal DNA quantification, which we developed here, correlated with the size of the necrotic area. Under our experimental conditions, the increase in fungal DNA leveled off between 3 and 4 dpi. Since we have no evidence for hyphal growth outside the necrotic area in leaf cells, we speculate that the vital fungus continues to grow in newly damaged cells but withdraws from the nutrient-depleted cells at the center of the necrotic area. This proliferation pattern may limit the time scale of the qRT-PCR approach to evaluate the disease incidence. Therefore, we recommend the pliman approach for longer incubation periods, whereas both methods, qRT-PCR and necrotic area determination, achieve accurate *B. cinerea* infection data for up to about 4 dpi.

While spore infection under many different conditions tested here did not lead to homogenous infection patterns, the robustness of the plug infection protocol was demonstrated by several independent repetitions. However, the increase in necrotic tissue varied among different independent runs of the experiments. Since *B. cinerea*-induced infections are favored in the temperature range from 15 °C to 25 °C [46], we speculate that this might have been caused by unattended temperature fluctuations in our greenhouse. It is also conceivable that differences in poplar metabolism under different greenhouse conditions affected fungal proliferation. These possibilities should be clarified by future investigations.

We conducted *B. cinerea* bioassays with different poplar species when the leaves were very young, right after bud break. Young leaves of strawberry plants or eucalypt trees were more susceptible to *B. cinerea* than old leaves [47, 48], while young leaves of bean plants were less susceptible [49]. The disease resistance of poplar leaves may also vary with leaf age and, thus, should be taken into account when using our *B. cinerea* infection protocol.

Our infection system is suitable for inducing strong damage to leaves in controlled experiments using vigorously growing *B. cinerea* hyphae. However, it should be noted that the natural infection caused by *B. cinerea* starts with spores landing on the plant surface [50]. The spores can germinate and penetrate the leaf, where they remain quiescent until favorable conditions prevail for a disease outbreak [5]. This behavior is also true for *B. cinerea* in poplar leaves because we isolated the *B. cinerea* strain, able to induce severe leaf lesions, from healthy-appearing leaves. This finding exemplifies the peculiarity of *B. cinerea* being able to remain symptomless within the host tissue, either in the quiescent stage [5] or by a putatively endophytic lifestyle [51]. In our study, *B. cinerea* also showed symptomless development when germinated from spores on poplar leaves, suggesting adaptation of this strain to *P.* x *canescens* and vice versa. The latent or asymptomatic infection strategy of *B. cinerea* highlights the importance of its molecular quantification.

The factors that lead to disease outbreaks and occurrence of necroses in poplar are still enigmatic. In *Arabidopsis thaliana*, the phyllosphere microbiome can grant resistance against *B. cinerea* [52]. Our results also hint towards a control of the phyllosphere microbiome on *B. cinerea* because disease outbreak required leaf surface sterilization. Since the microbiome of forest trees show seasonal fluctuations [53], its impact on the severity of *B. cinerea* infections may vary and thus, negatively affect experiments. However, it should also be noted that the ethanol treatment for leaf sterilization can alter leaf surface chemistry and thereby, putatively facilitate fungal infections. Therefore, further studies are necessary to distinguish these possibilities and clarify a potential protective role of the surface microbiome. Inside Arabidopsis leaves*, B. cinerea* secreted toxins induce programmed cell death before fungal proliferation [54, 55]. In the present study, photosynthetic integrity was diminished in areas adjacent to the necroses, although we could not find clear-cut support for fungal presence in these cells. Therefore, we speculate that *B. cinerea* sets off a cell lytic program similar to that in Arabidopsis, feeding on leaking cell contents. *B. cinerea* was also able to grow vertically through the leaf, evading through the open stomata and forming new conidia. Thus, our microscopic results provided novel insights into how *B. cinerea* colonizes poplar leaves.

The reduction of disease severity after methyl jasmonate treatment is in line with findings for other species like Arabidopsis or tomato [56, 57]. In poplar, methyl jasmonate treatment induces phenol-based defenses [58] and recruits a signaling cascade involving JAZ proteins, several transcription factors of the MYB and ERF families, and chitinases [59]. Collectively, these responses are known to play roles in the defense of Arabidopsis against *B. cinerea* [60–62]. The infection protocol described in the present work will enable researchers to investigate controls on this ubiquitous fungus and uncover conditions under which *B. cinerea* escapes the poplar defense systems.

Increasing our knowledge of necrotizing pathogens in tree species is important because fungal diseases threaten the health of our forests [63]. The generalist *B. cinerea* is a dangerous pathogen in climate change-stressed forests, causing increased disease incidences in drought-stressed trees [64]. Our bioassay will be particularly useful for translational research, transferring knowledge from the *B. cinerea* infection system in Arabidopsis [65] to poplar and in tree improvement programs using bioengineering to enhance tree resistance and screening the natural inter- and intra-specific variation of *B. cinerea* resistance in *Populus* sp.

## Conclusion

Here, we developed a novel bioassay for the poplar necrotizing pathogen *B. cinerea* and its molecular quantification in leaves. A major benefit of the method is that it is straightforward to use. The setup requires only plates with the *B. cinerea* strain, plastic bags, and photos of the necrotic area as the basis to quantify fungal disease severity. The bioassay is accomplished within 4 dpi, and an initial evaluation of the necrotic area can be done within an hour after image acquisition. The availability of an assay for the interaction of poplar with a necrotizing fungus will stimulate research in many areas, such as ectomycorrhiza-induced resistance [66], abiotic stresses [drought, heat, [67], nutrient stress [68]] or evaluation of new species for wood production [69]. Moreover, it also enables us to uncover molecular signaling pathways to combat necrotizing pathogens in an economically important tree species.

## Supplementary Information

**Additional file 1: Figure S1.** The effect of 70% ethanol poplar leaf surface sterilization on infection severity of *Botrytis cinerea *and mock-PDA plug inoculation at 4 dpi. **Table S1.** Origin of Poplar species challenged with *Botrytis cinerea*.

## Data Availability

Sequences are available at NCBI Genbank (https://www.ncbi.nlm.nih.gov/genbank/) under accession numbers ON740896 and ON740897. *B. cinerea* strain 261.71 from poplar is available in the public collection of DSMZ (https://www.dsmz.de) under the collection number DSM 114993.
