# Supplementary material for: Fast and easy bioassay for the necrotizing fungus Botrytis cinerea on poplar leaves
Source: Plant Methods. 2023 Mar 29;19:32. doi: 10.1186/s13007-023-01011-3 (PMC10061990; doi:10.1186/s13007-023-01011-3)
Supplement: Supplementary file 1 — Additional file 1: Figure S1. The effect of 70% ethanol poplar leaf surface sterilization on infection severity of Botrytis cinerea and mock-PDA plug inoculation at 4 dpi. Table S1. Origin of Poplar species challenged with Botrytis cinerea. [file 13007_2023_1011_MOESM1_ESM.docx]

**Additional information**

**Fast and easy bioassay for the necrotizing fungus *Botrytis cinerea* on poplar leaves**

Steven Dreischhoff^1^, Ishani Shankar^1^ Das, Felix Häffner^2^, Anna Malvine Wolf^3^, Andrea Polle^1^, Karl Henrik Kasper^1^*

* Correspondence: kkasper@gwdg.de

^1^ Forest Botany and Tree Physiology, University of Goettingen, 37077 Göttingen, Germany

^2^ Department Aquatic Ecosystem Analysis, Helmholtz Center for Environmental Research-UFZ, 39115 Magedeburg, Germany

^3^ Hessische Landesgesellschaft mbh, 35392 Gießen, Germany

**Additional Figure**


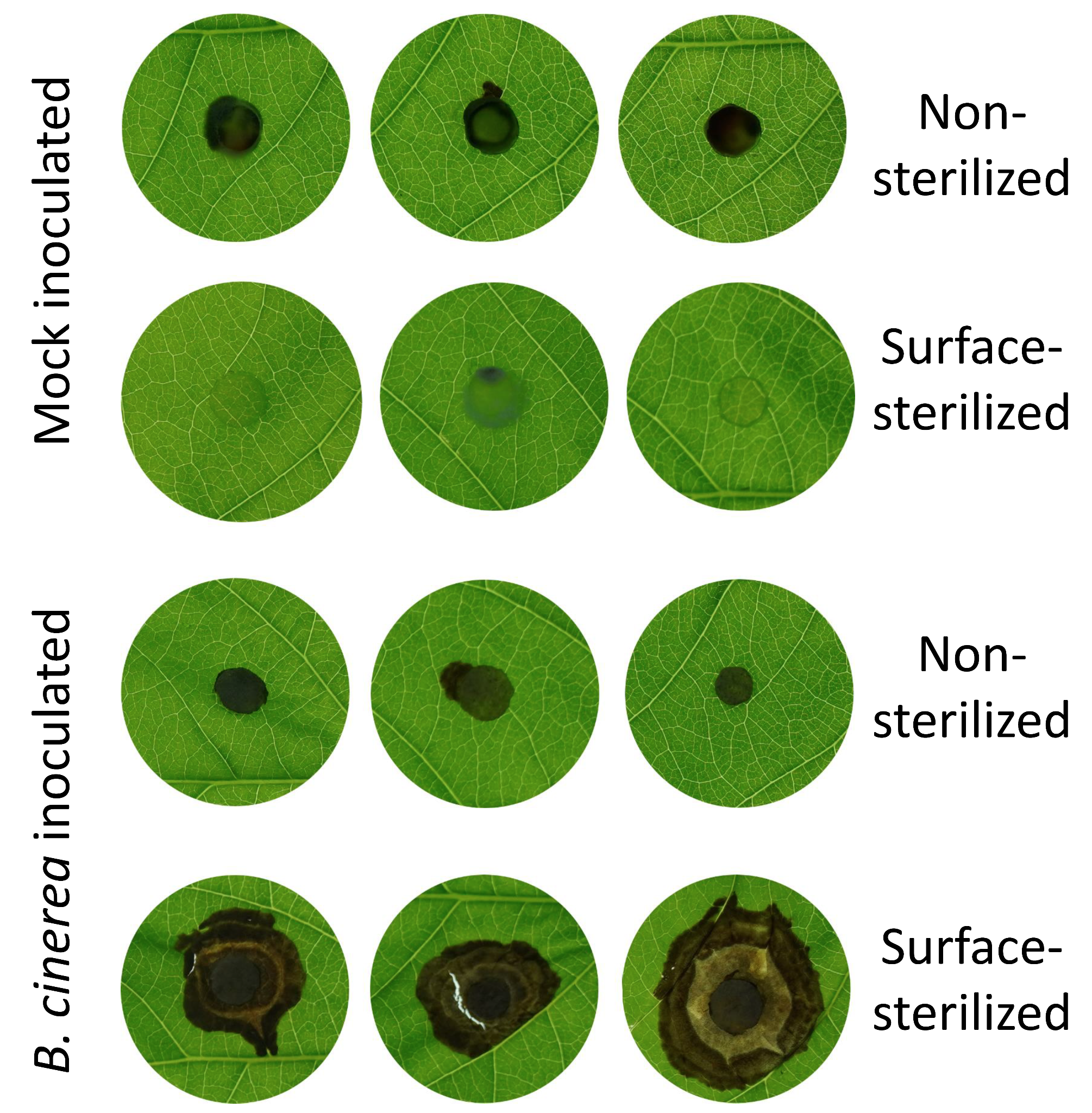


Supplementary Figure S1: The effect of 70% ethanol poplar leaf surface sterilization on infection severity of *Botrytis cinerea* and mock-PDA plug inoculation at 4 dpi.

Leaves of *Populus* x *canescens* were wiped three times with a 70 % ethanol-soaked paper tissue and subsequently inoculated with a sterile Potato-Dextrose-Agar plug (mock treatment) or a *Botrytis cinerea* plug from a seven-day-old Potato-Dextrose-Agar culture. The plugs were 6 mm in diameter. After the harvest, the plugs were removed and leaf disks (Ø=27 mm) were punched out around the previous position of the plugs.

**Additional Table**

**Additional** Table S1: Origin of Poplar species challenged with *Botrytis cinerea*.

| **Species** | **Section** | **Origin** |
| --- | --- | --- |
| *Populus alba* | *Populus* | Department of Forest Botany and Tree Physiology, University of Göttingen |
| *P.* x *canescens* | *Populus* | INRA 717 1B4, INRA, Nancy, France |
| *P. tremula* | *Populus* | NW-FVA^#^ Göttingen, formerly Hessische Forstliche Versuchsanstalt Hann Münden-Pappelkamp |
| *P. tremula* x *tremuloides* | *Populus* | Department of Forest Botany and Tree Physiology, University of Göttingen |
| *P. deltoides* | *Aigeiros* | NW-FVA Göttingen, formerly Hessische Forstliche Versuchsanstalt Hann Münden-Pappelkamp |
| *P. deltoides* spp. *Wislizeni* | *Aigeiros* | NW-FVA Göttingen, formerly Hessische Forstliche Versuchsanstalt Hann Münden-Pappelkamp |
| *P. nigra* | *Aigeiros* | NW-FVA Göttingen, formerly Hessische Forstliche Versuchsanstalt Hann Münden-Pappelkamp |
| *P.* x *canadensis* | *Aigeiros* | NW-FVA Göttingen, formerly Hessische Forstliche Versuchsanstalt Hann Münden-Pappelkamp |
| *P. balsamifera* | *Tacamahaca* | NW-FVA Göttingen, formerly Hessische Forstliche Versuchsanstalt Hann Münden-Pappelkamp |
| *P. candicans* | *Tacamahaca* | NW-FVA Göttingen, formerly Hessische Forstliche Versuchsanstalt Hann Münden-Pappelkamp |
| *P. cathayana* | *Tacamahaca* | NW-FVA Göttingen, formerly Hessische Forstliche Versuchsanstalt Hann Münden-Pappelkamp |
| *P. koreana* | *Tacamahaca* | NW-FVA Göttingen, formerly Hessische Forstliche Versuchsanstalt Hann Münden-Pappelkamp |
| *P. laurifolia* | *Tacamahaca* | NW-FVA Göttingen, formerly Hessische Forstliche Versuchsanstalt Hann Münden-Pappelkamp |
| *P. maximiwiwicziana* | *Tacamahaca* | NW-FVA Göttingen, formerly Hessische Forstliche Versuchsanstalt Hann Münden-Pappelkamp |
| *P. trichocarpa* | *Tacamahaca* | NW-FVA Göttingen, formerly Hessische Forstliche Versuchsanstalt Hann Münden-Pappelkamp |
| *P. simonii* | *Tacamahaca* | NW-FVA Göttingen, formerly Hessische Forstliche Versuchsanstalt Hann Münden-Pappelkamp |
| *P. suavaelens* var. Przewalksii | *Tacamahaca* | NW-FVA Göttingen, formerly Hessische Forstliche Versuchsanstalt Hann Münden-Pappelkamp |
| *P. wilsonii* | *Leucoides* | Botanical garden Kiel 2010 |
| *P. euphratica* | *Turanga* | clone B2 obtained from trees grown in the Ein Avdat region, Israel |

^#^ Niedersächsische Forstliche Versuchsanstalt
